# TRIM58 Interacts with ZEB1 to Suppress NSCLC Tumor Malignancy by Promoting ZEB1 Protein Degradation via UPP

**DOI:** 10.1155/2023/5899662

**Published:** 2023-01-05

**Authors:** Rongxin Shang, Jiakuan Chen, Yang Gao, Jijun Chen, Guoliang Han

**Affiliations:** ^1^Department of Thoracic Surgery, The Second Affiliated Hospital of Air Force Medical University, Xi'an, China; ^2^Department of Thoracic Surgery, The First Affiliated Hospital of Air Force Medical University, Xi'an, China

## Abstract

**Background:**

Currently, how to successfully control refractory and metastatic diseases remains a fundamental goal for clinicians to improve therapeutic effects for patients with non-small cell lung cancer (NSCLC). Several studies have discovered that TRIM58, a member of tripartite motif protein family, shows antitumor effect in multiple types of cancer. In this study, we aimed to further clarify the molecular regulatory network of TRIM58 and corresponding targets for NSCLC patients.

**Methods:**

TRIM58 expression in clinical tumor tissue samples and cancer cell lines was examined. Functional experiments including cellular invasion, cell metastasis, chemoresistance assay, and ubiquitination evaluation experiments were conducted to investigate the interaction between TRIM58 and ZEB1, which is a prime element of transcription factor network that controls epithelial-to-mesenchymal transition.

**Results:**

TRIM58 expression was characteristically decreased in NSCLC tumor tissues and cancer cell lines. Functional experiments demonstrated that TRIM58 suppression enhanced malignant biological behaviors including cellular survivability, migration, and invasion, as well as stem-like cellular phenotype of tumor cells. TRIM58 silencing also significantly enhanced the chemoresistance of NSCLC cells to chemoagents. TRIM58-ZEB1 interaction accelerated degradation of ZEB1 protein, thus further leading to the augment of tumor behaviors. Further detailed molecular experiments revealed that the interaction between TRIM58 and ZEB1 was mediated *via* ubiquitin-proteasome pathway (UPP).

**Conclusion:**

TRIM58 suppressed NSCLC through interacting with ZEB1 and promoting ZEB1 protein degradation *via* UPP. The present research sheds light on the interaction between TRIM58 and ZEB1, and TRIM58/ZEB1 axis might be the potential therapeutic targets of NSCLC.

## 1. Introduction

Lung cancer remains as one of the most deadly malignant diseases globally [1]. Among all lung cancer types, non-small cell lung cancer (NSCLC) occupies a major portion of patient numbers. As compared with other NSCLC subtypes, lung adenocarcinoma usually leads to worse prognosis [2]. Up till now, major progress has been achieved in lung cancer treatment, and novel advances in immunochemotherapy combined with surgical operations have enhanced the general prognosis of lung cancer patients. However, refractory and metastatic diseases are still imminent threats for lung cancer patients. For instance, nearly 20% of advanced NSCLC patients have reduced overall survival due to refractoriness of lung cancer disease [3]. Consequently, research should pay more attention to the underlying mechanism of lung cancer refractoriness and metastasis so as to further improve the efficacy of antitumor treatments.

Epithelial-mesenchymal transition (EMT) is a critical process in the development of tumor cellular invasion that converts polarized immotile epithelial cells to migratory mesenchymal cells. EMT has been intensively involved in tumorigenesis, progression, metastasis, and treatment refractoriness [4–6]. Researches utilizing immunodeficient mice model and lineage tracing techniques indicate that EMT is involved in tumor progression, which facilitates the capability of tumor cell self-renewal and differentiation into other types of tumor cells [7–9].

E-box binding zinc finger protein 1 (ZEB1) is a member of ZEB homeobox transcription factor family, located on human chromosome 10. ZEB1 is also a key transcription regulator involved in tumor progression [10]. It is abnormally expressed in many cancers and can promote migration, invasion, and metastasis of tumor cells. The enhancement of tumor cell plasticity is an important driving force for the progress of malignant tumors, and ZEB1 is the key factor mediating cell plasticity [11]. ZEB1 could promote tumor cell migration, invasion, and metastasis by changing cell plasticity and mediating EMT mechanism. It was reported that hypoxia-induced ZEB1 promoted cervical cancer progression via CCL8-dependent tumor-associated macrophage recruitment [12]. USP18 could promote tumor metastasis in esophageal squamous cell carcinomas via deubiquitinating ZEB1 [13]. The regulatory role of ZEB1 in NSCLC tumor malignancy has not been fully reported.

The ubiquitin-proteasome regulatory system controls protein homeostasis of almost the entire proteome [14–16]. Ubiquitin-mediated proteolysis primarily consists E1 activating enzymes, E2 conjugating enzymes, and E3 ubiquitin ligases. Accumulating evidences have found that E3 ligases of both the RING and the HECT family regulate EMT process via ubiquitination, including *β*TrCP, Skp2, Fbw7, and Cullin 7/Fbxw8 [17–21]. Tripartite motif proteins (TRIMs) belong to the family of E3 ubiquitin ligases. TRIM58, a member of TRIM family, containing a RING motif, a B box type 1 and 2, and a coiled-coil domain in its N-terminal region, has been identified as an E3 ubiquitin ligase and considered as an important regulator in several types of cancers [22]. However, the role of TRIM58 in NSCLC metastasis and progression, especially in cancer cellular EMT process has not been clearly elucidated. Therefore, in this study, we aimed to investigate the role and mechanism of TRIM58 in NSCLC disease progression and invasion. This research might provide novel clues in the management of metastatic disease in NSLCL treatment.

## 2. Materials and Methods

### 2.1. Patient and Sample Collection

Eligible NSCLC patients diagnosed in our facility from 2019/6/1 to 2020/6/1 were enrolled in this research. Patients with previous surgical treatment, radiotherapy, or chemotherapy were excluded. Tumor tissues and matched normal lung tissues were collected. Samples were stored in liquid nitrogen. Declaration of Helsinki was followed throughout experiments involving humans. The patients' informed consents were obtained. The conduct of this study was approved by the ethical reviewing committee of our institution. We followed the methods of Guo et al. [23].

### 2.2. RT-PCR

Sample RNA extraction was performed using RNAiso Plus agent (#9108, TaKaRa, Dalian, China). Then, reverse transcription PCR was used to acquire cDNAs. mRNA expression was subsequently detected using real-time PCR with SYBR green qPCR mix kit (#A301-01, Genstar). qRT-PCR reaction was performed under the following parameters: 95°C for 15 s, 57°C for 35 s, 73°C for 90s, with total cycles of 44. Primers were shown in the Supplementary Table 1.

### 2.3. Cell Lines

Tumor cell lines (NCI-H441/NCI-H157/NCI-H1975/NCI-H1395/NCI-H2087) and lung epithelial cell line (BEAS-2B) were acquired from American Type Culture Collection (ATCC, USA). RPMI-1640 (Gibco, USA) with 5% fetal bovine serum, 150 IU/mL penicillin with 150 *μ*g/mL streptomycin (Invitrogen, USA) were utilized for cell culture, under culturing parameter setting as 37°C, with 5% CO_2_.

### 2.4. Vector Construction and Cell Transfection

pLVX lentiviral vector was acquired as vectors for this research (Clontech, USA). Polymerase chain reaction (PCR) was performed using sequence templates of TRIM58 shRNA and TRIM58 and ZEB1 full-length cDNA sequences. Firstly, purification of PCR products were performed by 5% agarose gel, then products were treated by BamHI and EcoRI as well as empty pLVX vectors. And subsequent ligation was conducted using T4DNA ligase. Then, products were used for the *E.coli* DH5*α*—competent cells transformation. Afterwards, LB plate with ampicillin was utilized for culture overnight at 37°C. Vectors from positive clones were collected and sequenced (Invitrogen Biotech Co., Ltd). Then, all vectors were packaged and added into each treatment group with multiplicity of infection (MOI) of 15 and vectors titer was set as 1 × 10^8^/mL.

### 2.5. Western Blot

Cell group samples were purified using cold PBS (#C0221A, Beyotime, Shanghai, China). RIPA agent (#P0013B, Beyotime) was used for protein extraction. Then, BCA protein assay kit (#P0012, Beyotime) was applied to quantify protein content. SDS-PAGE with 12% separating gels were used for protein separation and samples were subsequently transferred onto PVDF membranes (#IPVH00010, Immobilon-P; Millipore, MA), which were incubated with primary antibodies overnight at 4°C after blocking in Tris buffer. The protein samples were incubated with the secondary antibody for 4 h. The antibodies used in this research were listed as follows: anti-E-Cadherin antibody (ab40772), anti-TRIM58 antibody (ab254768, Abcam, UK), anti-Vimentin antibody (ab92547), anti-beta actin antibody (ab8227), anti-N-Cadherin antibody (ab76011), anti-ALDH1A1 antibody (ab52492), anti-CD44 antibody (ab243894), and anti-ZEB1 antibody (ab203829).

### 2.6. Cellular Migration and Invasion Assay

Sample cells were inoculated with density of 5 × 10^4^ in the upper chamber (Corning, USA). Cells were incubated for 36 h with environmental parameter of 37°C, 5% CO_2_, and lower chamber were then fixed and stained for subsequent observation and quantification. A total of 12 randomly-chose fields per sample were selected for further quantification. The invasion experiment was performed with the same way except that the filters of the transwell chambers were coated with 30 mg matrigel (#4-ME-354262, BD Biosciences, USA).

### 2.7. Wound Healing

Cell samples were firstly plated into 6-well plate. Each plate was scraped by sterile 1 mL tip. Then, wounded plates were washed by PBS (#C0221A, Beyotime, Shanghai, China). Wound edge distances were calculated at three independent positions, and such measurement were repeated again 2 days postinoculation.

### 2.8. Cellular Survivability Assay

Multiple different concentrations of docetaxel, doxorubicin, and gefitinib were used to treat cell samples for 4 h, and afterwards, samples were purified using PBS and 75% ethanol was added in samples at -20°C to treat sample overnight. Subsequently, cells were incubated utilizing Annexin V-FITC (#331200, Thermo Fisher Scientific) and propidium iodide (PI) for 30 min in room temperature. Flow cytometric platform was utilized (FACScan™, BD Biosciences, USA) for quantification of apoptotic cells.

### 2.9. Coimmunoprecipitation (co-IP)

HEK-293 T, NCI-H441, and NCI-H1395 cells transfected with FLAG-tagged TRIM58 or HA-tagged ZEB1 were firstly treated by immunoprecipitation buffer. Afterwards, cell samples were incubated with 2 *μ*L FLAG/HA antibody and 15 *μ*L of Protein A/G magnetic beads (prewashed with lysis buffer) for 4 h in 4°C environmental setting. Subsequently, the beads were purified by wash buffer and target was eluted by 2× SDS loading buffer. Eluted proteins were treated under 95°C for 10 min before separation by SDS-PAGE and immunoblotting by FLAG/HA antibodies.

### 2.10. Immunofluorescence (If)

Cells were washed with PBS (#C0221A, Beyotime, Shanghai, China), and then paraformaldehyde was applied to fix cell samples for 20 min. Then, samples were permeabilized using 0.5% NP-40 for 30 min, and were blocked by 10% bovine serum albumin for 20 min. Afterwards, cells were treated with primary antibody for 3 h in room temperature, and were treated with fluorescein conjugated secondary antibody for another 2 h. Finally, DAPI and confocal microscopy were utilized to stain and perform imaging analysis on the slides, respectively. The antibodies applied in this research were listed as follows: anti-TRIM58 antibody (ab254768, Abcam, UK) and anti-ZEB1 antibody (ab203829, Abcam, UK).

### 2.11. CHX-Chase Assay

CHX-chase assay was performed using CHX (Selleck Chemicals). Cells were treated with 12.5 *μ*g/mL of CHX and the expression of ZEB1 and TRIM58 protein was measured via western blot at 0, 2, 4, and 8 h, respectively.

### 2.12. Ubiquitination Assay

Cells were transfected with ubiquitin (#UB-102H-1 M, Ubbiotech, Changchun, China) and TRIM58 overexpression vectors or shRNAs by jetPRIME (#101000046, Polyplus, France). Then, MG132 (30 *μ*M, #S2619, Selleck Chemicals, USA) was added for transfection (36 h). The samples were applied for western blot assay. Cell lysates were immunoprecipitated (IP) with the labeled antibodies and cultured overnight at 4°C. Finally, western blot was performed to measure the eluted proteins.

### 2.13. Establishment of Metastatic Tumor Model in Mice

All animal experiments have been approved by the Animal Care and Use Committee of the Second Affiliated Hospital of Air Force Medical University. Female athymic nude mice (6 weeks old) obtained from Beijing Vital River Laboratory Animal Technology (Beijing, China) were used in this research. The animals were divided randomly into two groups (*n* = 5 for each group). The mice in the group TRIM58 were injected with 5 × 10^6^ NCI-H441 cells transfected with TRIM58 overexpression vectors, the animals in the group vector were injected with 5 × 10^6^ NCI-H441 cells transfected with negative control vectors. Tumor cells were injected into the left armpit of mice. The tumor size was measured with vernier caliper, and the weight of mice was weighed. The drinking water, diet, mental state, and physical condition of the mice were observed. If the mice are found to have limping, extreme malaise, or cachexia, the mice shall be killed by decapitation and recorded. After 21 days, the animals were sacrificed, and the tumor of the mice was weighed.

### 2.14. Statistical Analysis

Statistical analysis was performed using SPSS (Version 21.0, IBM, USA). Results were shown as the mean value + SD with 3 independent experiments. Statistical analysis between two groups was performed by the Student *t*-test. ANOVA was used to analyze data among more than two groups. Pearson's test was performed to analyze gene correlation. *P* < 0.05 indicates statistical significance.

## 3. Results

### 3.1. Significant TRIM58 Downregulation in NSCLC Tissues and Cell Lines

**Figure 1:**
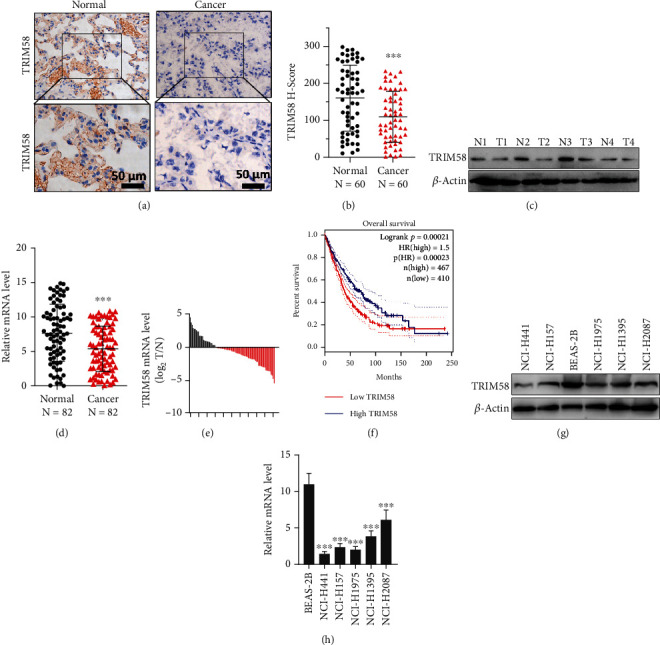
Significant TRIM58 downregulation in NSCLC was observed. (a) Differential expression of TRIM58 protein in NSLCL tumor tissue and normal lung tissue was analyzed with IHC staining. (b) H-Score comparison was performed on clinical samples of NSCLC patients retrospectively collected in our clinical center. (c) Western blot study on TRIM58 protein expression among four different NSCLC tumor tissues and adjacent normal lung tissue pairs. (d) qRT-PCR quantification of TRIM58 mRNA level between NSCLC tumor tissue and normal lung tissues. Samples were retrospectively collected in NSCLC patients receiving surgery in our clinical center. (e) Distribution of TRIM58 mRNA expression strength illustrated as Log2 value of expression ratio of tumor and matched normal lung tissue pairs retrospectively collected in our clinical center. (f) Overall survival comparison between NSCLC patient groups with high or low TRIM58 expression level. Log-rank test was utilized to examine the statistical significance. (g) TRIM58 expression among normal lung epithelial cell line BEAS-2B and NSCLC cancer cell lines including NCI-H441/NCI-H157/NCI-H1975/NCI-H1395/NCI-H2087 was measured with western blot. (h) qRT-PCR analysis was performed to measure the mRNA expression of TRIM58.

Firstly, we examined the protein expression of TRIM58 via IHC method in surgical samples of NSCLC tumor and matched adjacent normal tissues. As shown in Figure 1(a), TRIM58 was notably suppressed in cancer tissue, as compared with adjacent normal lung tissue samples. The average histochemistry score (H-Score) of NSCLC tumors (Figure 1(b)) was significantly lower than that of normal lung tissue (*P* < 0.001). Western blot assay in four paired-tumor and normal samples also supported the above finding (Figure 1(c)). Real-time PCR quantification suggested that mRNA expression of TRIM58 was significantly reduced in tumor tissue, compared with normal counterparts (Figures 1(d) and 1(e)).

To investigate the association of TRIM58 expression with the overall survival of NSCLC patients, log-rank test on overall survival differences between TRIM58 low- and high-expression patient groups was analyzed through GEPIA database. As shown in Figure 1(f), patients with high level of TRIM58 expression presented notably better overall survival period in comparison with patients with low TRIM58 expression level (*P* = 0.00021).

To validate the above findings, we further investigated TRIM58 mRNA and protein levels in NSCLC cell lines and normal human lung epithelial cell line. The results also suggested that NSCLC cell lines exhibited lower mRNA and protein level compared with normal lung epithelial cell line (BEAS-2B) (Figures 1(g) and 1(h)).

### 3.2. TRIM58 Suppression Was Correlated with Boosted Malignant Biological Behavior of NSCLC Cell Lines

**Figure 2:**
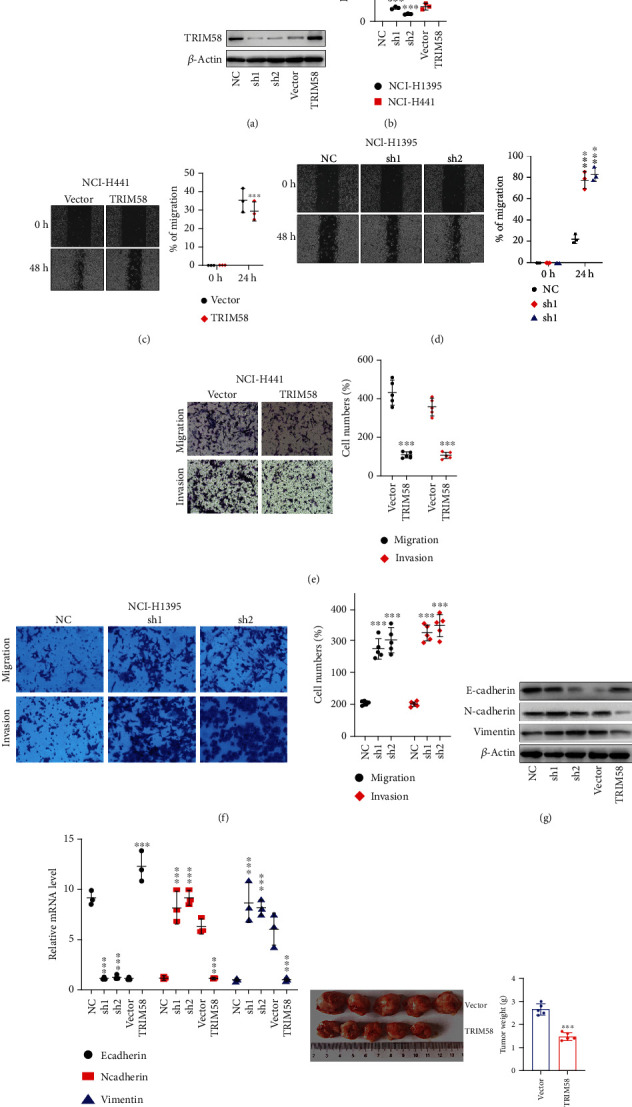
TRIM58 suppression was correlated with boosted malignant biological behavior of NSCLC cell lines. (a) Validation of expression modulation effects of TRIM58 specific shRNAs and overexpression vector via western blot in NCI-H1395 and NCI-H441 cancer cell lines. (b) The mRNA expression of TRIM58 was measured with qRT-PCR method. (c, d) Wound healing experiments on NCI-H441/NCI-H1395 cell lines transfected with TRIM58 overexpression vector/shRNAs or control vector. Percentage of migrated cells was calculated after 24 h. (e, f) Transwell experiments on NCI-H441/NCI-H1395 cell lines transfected with TRIM58 overexpression vector/shRNAs or control vector to quantify migrated cell number percentage. (g) Protein expression of EMT markers including E-Cadherin, N-Cadherin, and Vimentin were examined by western blot. Each cell group was treated by TRIM58 specific shRNAs or overexpression vectors. (h) The mRNA levels of E-Cadherin, N-Cadherin, and Vimentin were detected with qRT-PCR. (i) TRIM58 overexpression vector significantly inhibited tumor growth *in vivo* compared with group vector.

To investigate the role of TRIM58 in NSCLC cells, we performed expression modulation via TRIM58 shRNAs and overexpression vectors utilizing NCI-H1395 and NCI-H441 as cell models, respectively. The effects of shRNAs and overexpression vectors on TRIM58 level were validated via western blot and RT-PCR assay (Figures 2(a) and 2(b)). Next, we performed cellular migration and invasion assays on these two models. Wound-healing assay results suggested that TRIM58 overexpression significantly reduced cellular migration (Figure 2(c)), and TRIM58 shRNA treated tumor cells exhibited enhanced mobility (Figure 2(d)). Consistently, transwell assay confirmed that tumor cells with promoted TRIM58 expression demonstrated suppressed cellular mobility and invasiveness (Figure 2(e)), and vice versa (Figure 2(f)). Further molecular study indicated that the proinvasive tendency of tumor cells with TRIM58 suppression was probably related with mesenchymal phenotype transition of tumor cells, as western blot/qRT-PCR study both indicated that mesenchymal cellular markers (N-Cadherin and Vimentin) were significantly increased in TRIM58 silencing cell groups, while epithelial phenotypic marker (E-Cadherin) was considerably suppressed (Figures 2(g) and 2(h)). In addition, transfection with overexpression vector of TRIM58 markedly suppressed tumor growth *in vivo* (Figure 2(i)).

### 3.3. Association of TRIM58 with NSCLC Chemoresistance and Stem-Like Cellular Phenotype

**Figure 3:**
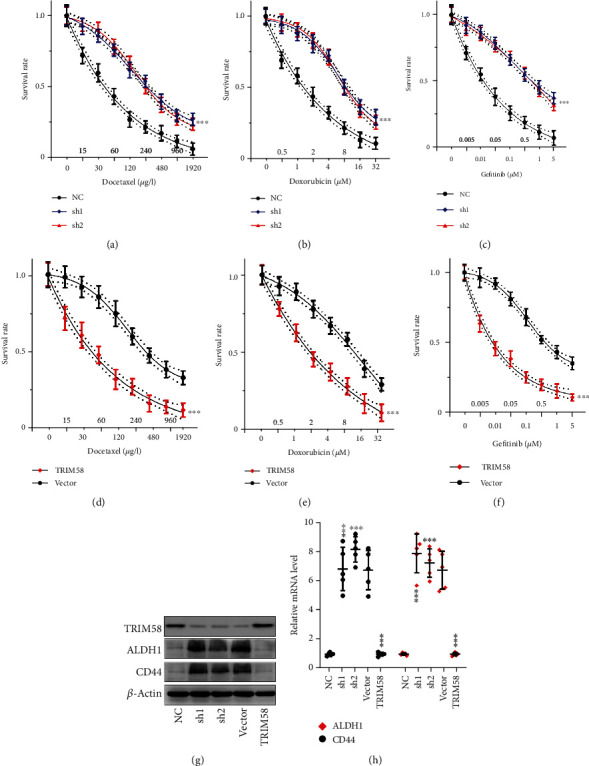
Association of TRIM58 with NSCLC chemoresistance and stem-like cellular phenotype. (a–c) After transfection with TRIM58 specific shRNAs or vector, the chemoresistance of NCI-H1395 cell lines treated with various concentrations of docetaxel, doxorubicin, or gefitinib were evaluated. (d–f) After transfection with TRIM58 overexpression vectors or control vector, the chemoresistance of NCI-H441 cell lines treated with various concentrations of docetaxel, doxorubicin, or gefitinib were measured. (g, h) Tumor stem-like cell marker detection via western blot/qRT-PCR methods, including ALDH1 and CD44. Cell line groups detected were treated with TRIM58 specific shRNAs/overexpression vectors/negative control vectors.

We evaluated the tumor cell chemoresistance of NSCLC by examining the survival rates of tumor cells after treated with chemoagents including docetaxel, doxorubicin, and gefitinib. TRIM58 silencing via sh1, sh2 (Figures 3(a)–3(c)), and control vector (Figures 3(d)–3(f)) could significantly enhance the chemoresistance of NSCLC cells to the three chemoagents (*P* < 0.001 for all comparisons). Western blot and RT-PCR experiments also suggested that TRIM58 silencing tend to increase the stem-like cellular phenotype of these tumor cells, as both protein and mRNA expression of stem-cell marker ALDH1 and CD44 demonstrated notably increase in tumor cells treated by TRIM58 shRNAs (Figures 3(g) and 3(h)).

### 3.4. Interaction between TRIM58 and ZEB1

**Figure 4:**
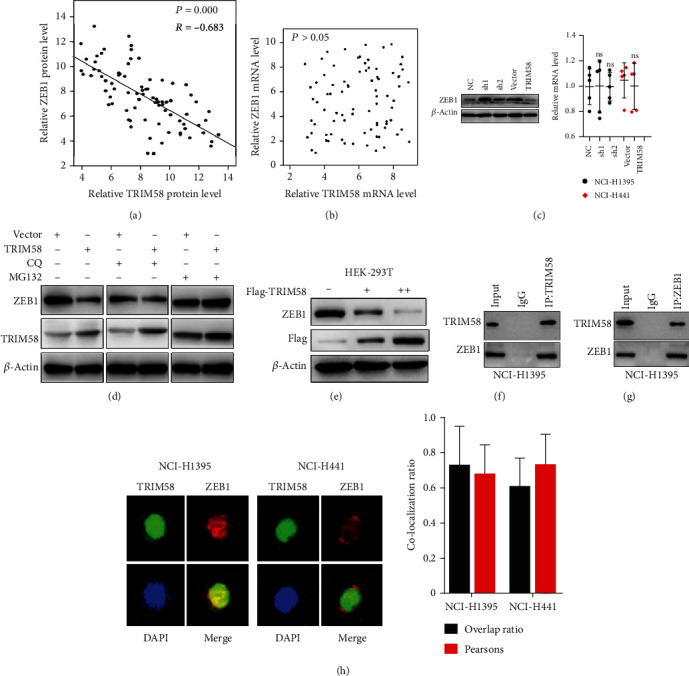
Interaction between TRIM58 and ZEB1 was identified. (a, b) Linear regression analysis was performed to evaluate the correlation between ZEB1 protein/mRNA expression and TRIM58 protein/mRNA expression. (c) Western blot and qRT-PCR were performed to analyze ZEB1 expression in NSCLC cell lines. Each experimental cell group was treated with TRIM58-specific shRNAs, overexpression vector, or negative control vector. (d) Evaluation of TRIM58 and ZEB1 protein expression on different tumor cell groups transfected with TRIM58 specific overexpression vectors, control vectors, CQ, or MG132. (e) Protein expression quantification of the association between FLAG-tagged TRIM58 and ZEB1 protein expression level in HEK-293 T cell line. (f, g) Coimmunoprecipitation study on the protein interaction of TRIM58 with ZEB1 in NCI-H1395 cell line. (h) Immunofluorescence experiment to investigate the colocalization of TRIM58 and ZEB1 in NCI-H441 and NCI-H1395 cell lines. Overlap ratio was subsequently calculated and compared between two different cell lines.

As ZEB1 functioned important role in NSCLC, we next investigated underlying connection between ZEB1 and TRIM58. Evidences from clinical sample experiments suggested that TRIM58 protein level was negatively correlated with ZEB1 protein level (Figure 4(a), *R* = −0.683, *P* < 0.001), while no significant correlation was detected between mRNA expression of TRIM58 and ZEB1 (Figure 4(b), *P* > 0.05). Subsequently, TRIM58 modulating experiments also suggested that TRIM58 overexpression or silencing showed no impact on ZEB1 mRNA expression, while TRIM58 silencing demonstrated significantly promotive effect on ZEB1 protein expression (Figure 4(c)). The above evidence suggested that posttranscriptional regulatory effects might be accounted for the impact of TRIM58 on ZEB1. Therefore, autophagy inhibitor (CQ) and proteasome inhibitors (MG132) were applied to investigate autophagy and ubiquitin-proteasome pathway (UPP) in ZEB1 modulation. As shown in Figure 4(d), TRIM58 overexpression cell group showed suppressed ZEB1 protein level when treated with CQ, while such effects vanished in MG132 treated group. In addition, by transfection of FLAG-TRIM58 vector, we found using HEK-293 T as model that ZEB1 protein expression was inhibited in dose-dependent manner (Figure 4(e)). Subsequent coimmunoprecipitation assay and immunofluorescence colocalization assay also demonstrated clues of TRIM58/ZEB1 protein-protein interaction (Figures 4(f)–4(h)).

### 3.5. Degradation of ZEB1 Was Promoted by TRIM58 Expression

**Figure 5:**
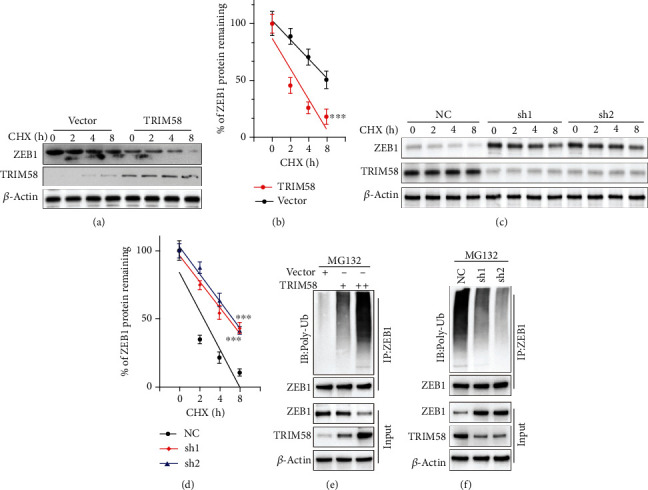
Degradation of ZEB1 was promoted by TRIM58 expression. (a, b) CHX chase experiment was conducted to measure UPP inhibition impact on TRIM58 and ZEB1 expression in NCI-H1395 cell line after treatment with TRIM58 overexpression vector or control vector. (c, d) CHX chase experiment was conducted to measure UPP inhibition impact on TRIM58 and ZEB1 expression in NCI-H441 cell line after treatment with TRIM58 shRNAs or negative control vector. (e, f) Co-IP was performed to detect the effects of TRIM58 modulation on ZEB1 protein expression in NSCLC cell lines. All cell groups were treated with MG132 to inhibit UPP, and each experimental group was additionally treated by negative control vector/TRIM58 shRNAs/TRIM58 overexpression vectors.

Next, we performed cycloheximide (CHX) chase assay to evaluate protein degradation velocity in tumor cells treated with or without TRIM58 overexpression vectors. As depicted in Figures 5(a) and 5(b), for cells treated with TRIM58 overexpression vector, protein degradation of ZEB1 was notably increased (*P* < 0.001), and vice versa (Figures 5(c) and 5(d)). Subsequent coimmunoprecipitation assay demonstrated that when treated with MG132, TRIM58 shRNA-treated cell group exhibited notably suppressed ubiquitination of ZEB1, resulting in the increased level of ZEB1 protein (Figure 5(e)). In comparison, for cells transfected with TRIM58 overexpression vectors, ubiquitination of ZEB1 was significantly promoted and ZEB1 protein level was significantly suppressed (Figure 5(f)).

### 3.6. Inhibition Effects of TRIM58-ZEB1 Interaction on NSCLC Tumor Behavior Were Observed

**Figure 6:**
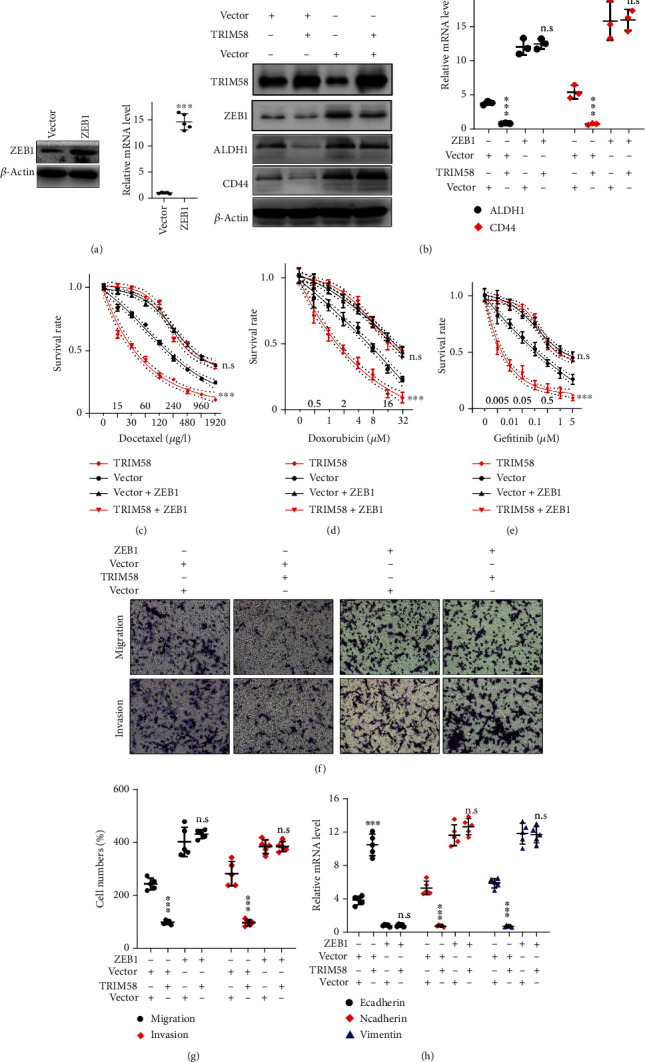
Inhibition effects of TRIM58-ZEB1 interaction on NSCLC tumor behavior were investigated. (a) Western blot and qRT-PCR were performed to investigate the impact of ZEB1 overexpression vector on ZEB1 expression. (b) Western blot and qRT-PCR experiment were performed to detect cancer stem-like cell markers including ALDH1 and CD44. Each experimental group was, respectively, treated with ZEB1 overexpression with or without TRIM58 overexpression vector. (c–e) Tumor cell line chemoresistance experiments were performed to detect NSCLC tumor survivability after treatment with different concentrations of docetaxel, doxorubicin, or gefitinib. Experimental cell group was, respectively, transfected with TRIM58 overexpression vector and/or ZEB1 overexpression vector. (f, g) Transwell experiment was performed to evaluate the effects of ZEB1 and/or TRIM58 overexpression vector transfection on NSCLC tumor cell invasion/migration. Percentage of migrated cells was calculated and analyzed. (h) EMT marker mRNA expression was measured with qRT-PCR after transfecting ZEB1 and/or TRIM58 overexpression vectors.

To evaluate the impact of TRIM58-ZEB1 interaction on the malignancy of lung cancer cells, ZEB1 specific overexpression vector was designed. The regulatory effects of ZEB1 overexpression vector on ZEB1 mRNA and protein expression were investigated (Figure 6(a)). We investigated the modulatory effects of independent or simultaneous transfection of ZEB1 and TRIM58 overexpression vectors on the expression of stem-like cell markers (ALDH1 and CD44). Our results demonstrated that the inhibitory effects of TRIM58 overexpression on ALDH1/CD44 expression can be reversed via combinatory transfection of ZEB1 overexpression vectors (Figure 6(b)). Furthermore, cellular chemoresistance assay also confirmed that transfection of TRIM58 + ZEB1 overexpression vectors reversed the promotive effects of solitary TRIM58 overexpression vectors on tumor cell sensitivity of chemoagent treatment (Figures 6(c)–6(e)). Moreover, subsequent cellular migration/invasion assay also provided consistent evidences that transfection of ZEB1 overexpression vector in combination of TRIM58 overexpression vectors abrogated the inhibitory effects of TRIM58 overexpression vector solitary transfection on tumor cell migration and invasion (Figures 6(f) and 6(g)). Detailed qRT-PCR assay on EMT related markers suggested that simultaneous transfection of ZEB1 and TRIM58 overexpression vectors also reversed the promotive effects of solitary TRIM58 overexpression vectors on E-Cadherin, and suppressive effects on N-Cadherin and Vimentin mRNA expression (Figure 6(h)).

## 4. Discussion

In this article, we demonstrated that TRIM58 suppression was a characteristic in lung cancer cells. And, further detailed experiments depicted novel mechanism that TRIM58 exhibited tumor inhibitory effects via interaction with ZEB1. Through binding with ZEB1 protein, TRIM58 enhanced ZEB1 protein degradation via ubiquitin-proteasome pathway so that TRIM58 was capable of minimizing ZEB1-mediated promotion of tumor migrated and survival capability. As reported in previous studies [24, 25], TRIM58 downregulation was associated with inferior prognosis and promoted malignancy in several tumors including gastric cancer, colorectal cancer, and lung cancer. As for the functions of TRIM58 in cancer pathogenesis and progression, previous studies have been suggested that TRIM58 takes part in the modulation of tumor microenvironment. As TRIM58 expression was positively correlated with M2-polarized macrophage and mast cells in KRAS-driven lung adenocarcinoma tumor tissues [26], which suggested that TRIM58 might facilitate the infiltration of immune cells in tumor microenvironment and enhance antitumor immune reaction. As a member of tumor suppressor genes (TSGs), previous research indicated that even in early stage of lung adenocarcinoma (LADC), aberrant promoter CpG island methylation resulted in robust expression suppression of TRIM58, which stimulated early carcinogenesis of LADC. Thus, it explained the potential mechanism of suppressed expression of TRIM58 in lung cancer, and such discovery also indicated that targeted aberrant TRIM58 methylation could be novel therapeutic target in future lung cancer treatment development.

**Figure 7:**
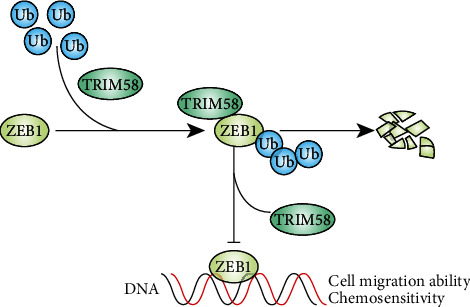
Schematic diagram of potential molecular mechanism of tumor inhibitory effects of TRIM58. TRIM58 interacted with ZEB1 and promoted ZEB1 protein degradation via UPP. Decreased ZEB1 promoted tumor cellular malignant biological behavior including cellular proliferation, migration, invasion, and chemoresistance.

ZEB1 protein consists of several protein binding domains including SMAD and p300 protein interaction domains. Researches have demonstrated that ZEB1 tightly control EMT process via transcriptional regulation, a series of genes involved in this process. Detailed study indicated that ZEB1 could inhibit cell polarity factors and basement membrane synthesis, while activate matrix metalloproteases expression including MMP-1, MMP-9, and MMP-14, which could enhance the remodeling of the basement membrane and facilitate tumor invasion into surrounding tissues [27]. And both in vitro and in vivo models have confirmed that overexpression of ZEB1 promoted invasive capability of multiple kinds of tumor cells. Moreover, beyond regulatory role of EMT, ZEB1 also participates in critical cellular functions which exerts great impact in tumorigenesis and tumor expansion. For example, Zeb1 knockout resulted in reduction of acinar ductal metaplasia and noninvasive pancreatic lesion [28], which supported the theory that ZEB1 was involved in early steps of pancreatic tumorigenesis. Our study provided novel theory of ZEB1 expression modulation by TRIM58 via UPP (Figure 7), indicating TRIM58-triggered ZEB1 depletion via UPP might also serve as a potential treatment strategy in NSCLC patients.

Indeed, the limitation of the *in vitro* model as well as retrospectively collected clinical samples utilized in our study warrant future consolidation experiments based on TRIM58-ZEB1 axis knockout animal models and clinical evidences based on multicentered perspective clinical trials. However, needless to say, the novel interaction between TRIM58 and ZEB1 may shed new light in NSCLC study and inspire future exploration in the potential significance in NSCLC patient treatment.

## 5. Conclusion

In this study, we discovered that TRIM58 was significantly suppressed in NSCLC clinical samples and tumor cell models. And, TRIM58 interacted with ZEB1 to facilitate ZEB1 protein degradation via UPP, which resulted in the exacerbation of NSCLC tumor expansion, invasion, and metastasis. Our study proposed for the first time that TRIM58-ZEB1 interaction might serve as potential target for future NSCLC treatment development.

## Data Availability

The data support this study could be requested from corresponding author.

## Abbreviations

NSCLC: Non-small cell lung cancer
EMT: Epithelial–mesenchymal transition
TRIM: Tripartite motif protein
UPP: Ubiquitin-proteasome pathway
PCR: Polymerase chain reaction
PBS: Phosphate-buffered saline
LADC: Lung adenocarcinoma.

## Supplementary Materials

Supplementary Materials Supplementary Table 1: primers used in this research.
