# Supplementary material for: TRIM58 Interacts with ZEB1 to Suppress NSCLC Tumor Malignancy by Promoting ZEB1 Protein Degradation via UPP
Source: Dis Markers. 2023 Jan 5;2023:5899662. doi: 10.1155/2023/5899662 (PMC9836804; doi:10.1155/2023/5899662)
Supplement: Supplementary Materials — Supplementary Table 1: primers used in this research. [file 5899662.f1.docx]

| **Supplementary Table 1** Primers used in this research | | |
| --- | --- | --- |
| Gene | Forward/Reverse | Sequence |
| TRIM58 | Forward | 5’- AGTCCTGAGCAGAAGTAAGGC |
| TRIM58 | Reverse | 5’- GGATCCAGCTTTACATCCAC |
| ZEB1 | Forward | 5’- GGCATACACCTACTCAACTACGG |
| ZEB1 | Reverse | 5’- TGGGCGGTGTAGAATCAGAGTC |
| ALDH1 | Forward | 5’- GGAATACCGTGGTTGTCAAGCC |
| ALDH1 | Reverse | 5’- CCAGGGACAATGTTTACCACGC |
| CD44 | Forward | 5’- CCAGAAGGAACAGTGGTTTGGC |
| CD44 | Reverse | 5’- ACTGTCCTCTGGGCTTGGTGTT |
| E-Cadherin | Forward | 5’- GCCTCCTGAAAAGAGAGTGGAAG |
| E-Cadherin | Reverse | 5’- TGGCAGTGTCTCTCCAAATCCG |
| N-Cadherin | Forward | 5’- CCTCCAGAGTTTACTGCCATGAC |
| N-Cadherin | Reverse | 5’- GTAGGATCTCCGCCACTGATTC |
| Vimentin | Forward | 5’- AGGCAAAGCAGGAGTCCACTGA |
| Vimentin | Reverse | 5’ - ATCTGGCGTTCCAGGGACTCAT |
| GAPDH | Forward | 5’ -GTCCATGCCATCACTGCCAC |
| GAPDH | Reverse | 5’ -AAGGCTGTGGGCAAGGTCAT |
